# Mechanisms of overdose from unintentional fentanyl use when intending to use stimulants: a qualitative study of people who use stimulants and avoid opioids

**DOI:** 10.21203/rs.3.rs-8555473/v1

**Published:** 2026-02-17

**Authors:** Bryson O Gomez, Elyssa Y Samayoa, Phillip O Coffin, Leslie W Suen, Susan L Ivey, Kelly R Knight, Caravella McCuistian, Ayesha Appa, Alexander R Bazazi

**Affiliations:** University of California, San Francisco; University of California, San Francisco; University of California, San Francisco; University of California, San Francisco; University of California, San Francisco; University of California, San Francisco; University of California, San Francisco; University of California, San Francisco; University of California, San Francisco

## Abstract

**Background:**

As many as two-fifths of nonfatal opioid overdoses in San Francisco, CA are from unintentional fentanyl use, with most people intending to use stimulants. However, the mechanisms of fentanyl exposure are unclear.

**Objective:**

To characterize mechanisms of unintentional fentanyl use resulting in non-fatal overdose among people who intend to use stimulants and not opioids.

**Methods:**

We interviewed 17 people using stimulants and avoiding opioids who experienced an overdose attributed to unintentional fentanyl use between August 2023 and July 2024. Interviews were semi-structured and included questions about drug use practices, the events leading up to the most recent overdose, and ways participants protected themselves from fentanyl. Informed by the risk environment framework, we used an inductive coding approach to identify key themes, characterizing the social contexts and mechanisms of unintentional fentanyl use and non-fatal overdose.

**Results:**

Participants reported using methamphetamine and cocaine in powder and rock/crack form and reported smoking, injecting, or intranasal use. We identified three distinct phases of drug use in which participants were exposed to fentanyl they thought to be a stimulant: procurement, storage, and consumption. Participants employed a range of practices to protect themselves from fentanyl, but their state of mind and social context created situations in which usual precautions were ineffective or not utilized. Structural factors, including inadequate housing, criminalization of sex work and drug use, and unpredictability in drug supply sources, contributed to overdose from unintentional fentanyl use.

**Conclusions:**

We document multiple mechanisms of overdose from unintentional fentanyl use which may operate at individual, interpersonal, and/or structural levels. The complexity of the phenomenon, which cannot be fully explained by “contamination” of stimulants or any other single mechanism alone, suggests interventions are needed at multiple levels of the risk environment to prevent overdoses among people who use stimulants. A combination of policy changes to address structural factors and implementation of individual-level harm reduction practices is required to address overdose from unintentional fentanyl use.

## 1. Background

In 2024, the opioid overdose crisis claimed the lives of over 54,000 people in the US^1^ and 495 people in the City and County of San Francisco.^2^ Overdose fatalities are largely driven by fentanyl, which now accounts for over 95% of opioid overdoses in San Francisco.^2,3^ Although the majority of fatal overdoses involve fentanyl and other drugs,^2,4^ the intentionality of fentanyl use among these overdoses is unclear. Many individuals intentionally use multiple drugs concurrently, yet concerns about unintentional exposure to fentanyl are increasing among people who use drugs, harm reduction service providers, and substance use researchers.^5–9^ Exposure of people who use heroin to fentanyl, either through adulteration of heroin or substitution, is well-documented.^10–12^ However, less is known about opioid overdose vulnerability resulting from unintentional fentanyl use among people who intend to use non-opioid substances.

Our previous research found that more than one in three people experiencing a nonfatal opioid overdose managed by paramedics in San Francisco reported no intentional opioid use, with most of these individuals intending to use a stimulant (cocaine or methamphetamine).^13^ Overdose from unintentional fentanyl use among people intending to use stimulants may be widespread: clusters of fentanyl overdoses have been documented among people who reported intending to use stimulants,^14,15^ national drug seizure and community drug testing data demonstrate the presence of fentanyl in stimulant samples,^16,17^ and high rates of fentanyl positivity have been found in urine and hair samples from people who use stimulants and other non-opioid substances.^18,19^

Qualitative research with people who use stimulants has documented concern over an increased risk of fentanyl overdose, fentanyl being present in other drugs, frequent personal exposure to fentanyl, and personal stories of unintentional fentanyl use through suspected adulteration of stimulants or through cross-contamination of smoking equipment.^6,7,20,21^ Recent research hypothesized three main pathways attributed to opioid-stimulant involved overdoses, either through accidental exposure to opioids among people who use stimulants, intentional use of opioids by people who use stimulants but lack opioid tolerance, or people who use opioids beginning to use stimulants to counteract sedation from fentanyl.^9^

Other studies have examined unintentional fentanyl use from the perspective of fentanyl contamination of stimulants.^20^ Drug testing data from San Francisco and elsewhere have rarely or only intermittently detected fentanyl in samples sold as stimulants, and it is unclear whether fentanyl found in stimulant samples represents trace contamination or sufficient quantities to cause opioid overdose,^22–25^ raising questions about the mechanisms of fentanyl exposure among people unintentionally using fentanyl.

We conducted a qualitative study to examine the pathways through which people who use stimulants and avoid opioids experienced an overdose from unintentional fentanyl use. A better understanding of the mechanisms of exposure to fentanyl among people who use stimulants is necessary to develop evidence-based overdose prevention interventions for this population.

## 2. Methods

### 2.1. Study Setting

Participants were primarily recruited from a post-overdose outreach team. In San Francisco during the time of the study, individuals who had experienced a non-fatal overdose and received paramedic intervention were referred to one of several post-overdose outreach teams to facilitate linkage to care. The Home Outreach Prevention and Engagement (HOPE) team outreached to housed individuals who had experienced a non-fatal overdose and was the primary source of recruitment. Most HOPE clients were housed in Single Room Occupancy (SRO) residential hotels,^26^ which in San Francisco are privately owned or acquired by the city or non-profit agencies to provide affordable housing, with tenants living in private rooms, often with shared public bathrooms; some sites offer on-site case management as well as other health and social services.^27^ Nearly all study participants resided in the South of Market and Tenderloin neighborhoods of San Francisco, which have the highest concentrations of drug overdose in the city.^2^ These neighborhoods also have previously been the targets of discriminatory policies, such as redlining, and include a higher concentration of Black residents compared to the citywide average.^28^

### 2.2. Recruitment

This study was approved by the University of California, San Francisco Institutional Review Board (#23–38546). We recruited adults who use stimulants and had experienced an overdose from unintentional fentanyl use that required naloxone administration in the past 120 days. We excluded individuals who intentionally used opioids, even intermittently. Participants were referred to the study by the HOPE team, other harm reduction providers and clinicians, or other participants in the study. This recruitment strategy resulted in a racially and ethnically diverse study sample. Recruitment continued until thematic saturation was reached.^29,30^

### 2.3. Interviews and Data Collection

BG, AB, and ES conducted qualitative interviews between August 2023 and July 2024 using a semi-structured interview guide eliciting details of participants’ recent overdose to understand mechanisms of unintentional fentanyl use and strategies participants employed to prevent opioid overdose. Interviews lasted 40 to 60 minutes. A $50 Visa gift card was provided for participation.

### 2.4. Qualitative Data Analysis

Interviews were recorded, transcribed, de-identified, and imported into ATLAS.ti (version 24.1.0) for coding and analysis. We used an inductive coding strategy to identify codes and themes related to events leading up to the overdose and micro- (e.g., group dynamics, mindset, and setting of drug use) and macro-level factors (e.g., drug and housing policy, structural violence) that contribute to overdose risk.^31–33^ Coders (BG and ES) met weekly with the principal investigator (AB) to discuss findings, iteratively refine the codebooks based on broad topics from the interviews, memos, and analytic discussions, and discuss disagreements until consensus was reached, with the principal investigator facilitating resolution. The narrative of the overdose and events leading up to the overdose were analyzed to identify and contextualize mechanisms of unintentional fentanyl use. Informed by ethnographic work documenting and conceptualizing the complex behavioral and social practices involved in injection drug use,^34^ we attempted to discern the temporal sequence of drug use behaviors with attention to micro-level material practices and their social context.

## 3. Results

We interviewed 17 participants a median of 19 days after they experienced an overdose from unintentional fentanyl use. All participants’ drug of choice was a stimulant, either powder cocaine, crack cocaine, methamphetamine, or a combination. Most participants identified as being from a racial and ethnic minoritized group, and most identified as male (Table 1). 35% of participants reported currently using stimulants via injection with 53% reporting lifetime history of injection drug use. The most common route of administration for stimulants was smoking (94% of participants), followed by injection (35%) and intranasal (24%), with many participants reporting using more than one route of administration. The reported route of administration during participants’ most recent overdose was 53% smoking, 24% injection, 18% intranasal, and 6% oral.

We found varied pathways to unintentional fentanyl use occurring along the sequence of behaviors from drug procurement, storage, preparation, and consumption. Fentanyl was ultimately unknowingly introduced to participants when they obtained, stored, or consumed what they thought was a stimulant. Participants reported relying on a set of risk reduction practices to prevent themselves from unknowingly using fentanyl, but these practices failed or were not employed at the time of overdose. In sum, unintentional fentanyl use involved two intersecting processes: (1) the introduction of fentanyl to the participant through drug procurement, storage, or preparation and consumption; in combination with (2) the failure of risk reduction strategies to avoid fentanyl use. Participants’ ability to adhere to their typical risk reduction practices was influenced by their state of mind, the interpersonal context of drug use at the time of overdose, and structural factors such as the unpredictable drug supply and safety in housing.

### 3.1. Mechanisms of Unintentional Fentanyl Use

#### 3.1.a. Drug Procurement

Some participants unintentionally obtained fentanyl while believing they were obtaining a stimulant. This often occurred when participants found an unidentified drug that they assumed or hoped was a stimulant, or when they obtained their drug from an unfamiliar source. After experiencing an overdose, participants then concluded that the drug was in fact fentanyl or had been contaminated with fentanyl, with fentanyl avoiding detection due to its similar appearance to stimulants. Interestingly, while many participants spoke about the phenomenon of fentanyl being mixed with stimulants, only one participant attributed their personal overdose to fentanyl contaminating or being mixed with their stimulant. The dominant narrative was that after overdosing participants believed they had used fentanyl alone in place of a stimulant. One participant described how he overdosed after using a drug that he found in a shared bathroom in his SRO:

Basically one of my friends found a bag of what we thought was coke in, in a bathroom and, and so, you know…I hit it, it seemed like, it seemed like it started to crackle like, like crack but, but then all of a sudden, that’s the last thing I remember, is just hitting it and just, and, and then the next thing I know…my two friends are looking at me saying, “Are you okay, [Participant], are you okay?” And there was nothing I could do, I was just fading out.

− 47-year-old multiracial man

Another participant was able to contact the supplier of her drug after the overdose to confirm that she was accidentally sold fentanyl instead of crack cocaine; when she called, she learned that he had also overdosed on fentanyl:

The person that I got it from when I OD’d, he didn’t know that it had fentanyl… he had sold my baby daddy some, he gave my baby daddy some fentanyl…I didn’t know that, that they had gave it to me, too. So my kid’s papa told me to tell [the supplier] … he said, “Tell [the supplier] he gave you fentanyl.” So I called, I said, “My baby daddy said you gave him some fentanyl.” [The supplier] said, “I might have, I didn’t do it intentionally,” because he sells fentanyl, too, so, and crack and weed, but he didn’t know that he, he had mixed it, so I guess it was an accident. So I called and told him what had happened to me, too, and he said he didn’t do it intentionally, if he did he apologized. But he had also OD’d so…

− 45-year-old Black woman

#### 3.1.b. Drug Storage

Unintentional fentanyl use occurred as a result of storing stimulants together with fentanyl, either knowingly or unknowingly. One way this occurred was by storing fentanyl and stimulants in the same space, either in syringes, bags, or as loose crystals, and not being able to tell one drug from another. One participant discussed how he bought a rock of crack cocaine for himself and a rock of fentanyl for somebody else and then stored them together in his pocket. He uses the term “iso,” which is typically used to refer to a more potent fentanyl product in San Francisco (with local data not showing the presence of isonitazenes in the drug supply).

##### Participant

I was buying that iso for a lady…I [already] had crack…but I had it in the same pocket [as the iso].

##### Interviewer

Okay, so you put the iso and your crack in the same pocket.

##### Participant

And that’s, that was not smart.

− 73-year-old Black man

When the participant then wanted to use his crack cocaine, he wasn’t able to tell which substance was the fentanyl and which was the crack cocaine. He then ineffectively tried to differentiate the two by biting one of the rocks, stating, “I always taste crack before I use it… and then I’ll just wait for a minute or two to see what it does.” This uncertainty in drug identity after storing the two drugs together ultimately led to him overdosing on fentanyl.

Drug storage related exposure occurred when participants stored their stimulants in equipment previously used to store fentanyl and mixed fentanyl with their stimulant in the process. This participant described how he accidentally introduced fentanyl residue into his methamphetamine by storing his drugs in a tin that his friend had used to store fentanyl:

##### Interviewer

Why do you think this overdose happened?

##### Participant

Because I’m an idiot, and I took a tin from a friend that was going to throw it away, and I didn’t clean it. I didn’t think that it was going to have fentanyl in it, which, I tested it after [the overdose], and it did.

− 4*4-year-old White man*

#### 3.1.c. Drug Preparation and Consumption

Other participants were introduced to fentanyl during the process of consuming a stimulant. These individuals often were smoking a stimulant alongside someone who was smoking fentanyl out of an identical pipe, which allowed for their equipment to get mixed up. Multiple participants noted that the same kind of smoking equipment is used for smoking both methamphetamine and fentanyl, making it impossible to distinguish whether smoking equipment contains one substance or the other.

Yeah, …and [my friend] put [fentanyl] in a separate bong and it was the same fucking looking bong and … I just picked it up and took a bong hit of it and I woke up at fucking [hospital]! Like, “This is the fentanyl and this is the speed one,” and I’m like, “Whatever,” and I was playing drums and I just grabbed the wrong one and just, that’s all I remember.

− 47-year-old multiracial man

Unintentional fentanyl use happened as a result of someone intentionally adding fentanyl to a participant’s pipe when the participant was smoking a stimulant with the intention of causing an overdose. One unhoused participant was smoking crack cocaine and let a stranger use her pipe, after which she overdosed and awoke to find her personal items missing:

I was smoking, I let this guy use my pipe and he put some fentanyl on there. I didn’t know what it was but I started feeling kind of funny. Next thing I know I woke up, I was in an ambulance. And they took me to the hospital and they checked me out and everything. So basically what he was trying to do was steal my credit cards and he took, he took my food stamp card and my ATM card.

− 65-year-old Black woman

This participant’s story demonstrates how being unhoused and not having access to a safer place to use drugs can increase vulnerability to overdose from unintentional fentanyl use in this manner.

### 3.2. Failure of Risk Reduction Strategies Used by Participants

Participants employed a variety of risk reduction strategies to protect themselves from fentanyl exposure, with two common strategies being sensory inspection of drugs and avoidance of fentanyl and people who use fentanyl. However, these strategies are difficult to implement in the fentanyl era, potentially leading to unintentional fentanyl use.

#### 3.2.a. Fentanyl and Stimulants are Indistinguishable

Participants relied on using multiple senses to determine the identity of the drug they were using as their primary method of reducing risk of fentanyl exposure. Participants described using the appearance, texture, taste, and smell of a drug to distinguish their stimulant of choice from fentanyl. However, the utility of this method was limited as fentanyl was often indistinguishable from stimulants.

I forgave that person [that gave me the fentanyl I overdosed on] because he didn’t know because he was buying [fentanyl] from the dealer. Saying it was, because you can’t decipher between meth and fentanyl now because it’s all powder form.

− 46-year-old Native American and Hispanic man

Another participant similarly visually inspected his drug prior to overdosing, since he was with people who use fentanyl. However, he was misinformed about the visual appearance of fentanyl, believing fentanyl was always colorful instead of white. This led to false reassurance before using the drug:

So when I went to go do what I was going to do… it didn’t even register to me that, that, oh, this might [be fentanyl]…because the bag that I had my stuff [methamphetamine] in looked exactly like the bag that they had their stuff [fentanyl] in… I thought fentanyl was like pinkish, or was like multicolored or whatever.

− 44-year-old Black man

#### 3.2.b. Fentanyl is Difficult to Avoid

Another significant risk reduction method utilized by many participants was avoiding fentanyl altogether, including seeking drug suppliers that only sell stimulants and not allowing people who use fentanyl into their living space as a means of protecting themselves. However, some participants described difficulty in carrying out this practice as a result of the saturation of the local market with fentanyl.

I’ve noticed that you can only get, well, I mean, you can get meth but you can only get fentanyl, like you can’t get black tar heroin, I mean, to save your life. And not that I’m looking for it, but my friend was talking about that the other day and he said that he was trying to get that for someone and they couldn’t find it anywhere. So it’s all, it’s, the, the market has been completely saturated with fentanyl.

− 52-year-old White man

Additionally, some participants would relax their precautions and allow people to use fentanyl in their space in order to help another. Participants would do this when acquaintances who use fentanyl did not have a safe space to use fentanyl.

**There wasn’t really anything unusual about that day [that I overdosed] except that… this guy came over who I don’t normally** hang out with, we were kind of just getting to know each other. I met him through this other friend of mine and then he’s living at a…it’s not a shelter but it’s sort of like a shelter, and so I felt bad for him because, you know…he doesn’t have keys, like he just, he’s kind of in a controlled environment and so I felt bad for him so I let him come over and we were just watching TV and smoking, and he was doing fentanyl and I was doing meth, and then that’s when it went haywire.

− 52-year-old White man

This scenario demonstrates how restrictive housing policies can influence where and with whom people use drugs and constrain options for safer use. In the absence of other safe spaces for drug use, community members with less regulated housing, including the participant above, fill that need. By doing so, people who normally protect themselves by keeping fentanyl out of their living space may forego this risk reduction strategy, which creates the opportunity for mixing up fentanyl and stimulants.

### 3.3. State of Mind Influences Risk Reduction

Participants’ motivations for using drugs and their state of mind influenced their willingness and capacity to enact risk reduction strategies, leading to forgoing usual risk reduction practices or ignoring sensory cues that could have indicated that a drug was fentanyl rather than a stimulant. The participant mentioned above, who experienced an overdose after storing his methamphetamine in a tin previously used by a friend who uses fentanyl, described how fatigue and frustration prevented him from noticing the potential fentanyl contamination from the tin:

I mean, I, when it happened it was my own stupid mistake, it, I never do that, like if I see a tin…I usually don’t take the tin, like, but I’m going to clean it or I would have put one and one together that it was [friend who uses fentanyl]’s, you know, I didn’t do, like I didn’t make that connection because I was just frustrated at the time, tired.

− 52-year-old White man

Some participants described how intoxication from other drugs contributed to their overdoses.

And I remember that day, it looked too white, too white to be true what it was because crystal meth is just like glistening, but this was a white form. Why did I take it? Because I was drunk and I was like, you know, and next thing you know I was on my way to the hospital and I was like, it was just, it was just tripped out.

− 46-year-old Native American and Hispanic man

An internal sense of urgency to use can reduce the use of risk-reduction methods. This urgency can be stem from a desire to get high or reduce withdrawal symptoms, or a need for energy to work. For one participant, who is a sex worker, a combination of withdrawal from stimulants and an immediate need to use so that she could work contributed to her using a drug with an unknown identity provided by a potential client that ended up being fentanyl:

…me and this guy were just talking and smoking a joint and he said, “Oh, I have some cocaine. Actually, I’m not sure if it’s cocaine or fentanyl,” and I looked at it, I said, “I think it’s cocaine.” And I’d been up for a while on meth and so it wasn’t working for me, I was like saturated, I couldn’t really do any more of that [methamphetamine] so I was like, oh, yeah, I really want to snort a line of coke. And I had to get rent money, too, so I was like trying to stay awake, okay. So I snorted a big line of it, or like, like the size of my pinky nail, you know, of cocaine, well, what I thought was cocaine. It turned out to be [fentanyl].

− 51-year-old White woman

This participant went on to situate the urgency of her use preceding the overdose in the context of how Supplemental Security Income (SSI) policies and the high cost of living limited her options to meet basic needs:

[Using methamphetamine] also has to do with the kind of lifestyle you end up in when you have to pay sixteen hundred dollars in rent and you only get nine hundred dollars a month…for all of your expenses… Everything included in your SSI isn’t enough to survive on in San Francisco. There’s no way, so you have to supplement your income somehow, and unless you steal or you deal drugs or you can get a job and not get caught by SSI you’re going to end up doing what I did, which was prostitution. And so it’s, it’s really challenging to stay out of crime when you can’t have a W-2 or lose your benefits.

### 3.4. Interpersonal Relationships Influence Risk of Unintentional Fentanyl Use

Participants’ willingness and ability to enact their usual risk reduction methods were influenced by their interpersonal relationships. For example, participants who usually maintained a fentanyl-free living space as a risk reduction method allowed fentanyl into their space either due to a desire to help another in a time of material need or from peer pressure. One participant discussed how he finds it difficult to maintain his fentanyl-free space as other people in his social circle use fentanyl, and he feels pressured into letting them in, providing a safer drug use environment for people who otherwise may not have a safe space to use fentanyl:

And that won’t be that hard because there aren’t that many of those people [who use fentanyl] and they’re not really my friends anyway, it’s just that sometimes my friends who are friends with those people will show up at my door with them. And that can be uncomfortable, but I can, I think I’m strong enough now to say, you know, “I had an overdose and I don’t allow fentanyl in my apartment, that’s it, so your friend has to go.” I mean, I think I’m, I think after what happened it gave me some strength… Because… [the overdose] wasn’t fun.

− 52-year-old White man

Some participants were unable to enact risk reduction practices for themselves as they relied on others to obtain or prepare drugs for them, so they instead had to rely on others to reduce risk for them. One participant described how she relied on her boyfriend, who used fentanyl and stored her methamphetamine alongside his fentanyl, to inject her with methamphetamine since she was unable to inject herself.

#### Participant

My boyfriend at the time, he did fentanyl, so like, so I, we got up in the morning and he was trying to hit me, and like usually he does all, he would do it all the time, like, and he would always, and never have a problem, like there would never, he would never miss or anything like that or, you know, just ‘jing jing’ and get it done, you know what I’m saying?

#### Interviewer

Do you also hit yourself?

#### Participant

No, I can’t hit myself…I don’t know how to.

− 36-year-old mixed-race Black woman

This gendered power differential led to the participant relying on her boyfriend to reliably distinguish between fentanyl and methamphetamine, constraining her agency to deploy strategies to reduce her risk of fentanyl overdose.

## 4. Discussion

Our study provides unique insight into the causes of overdose from unintentional fentanyl use by being one of the first to specifically interview people who use stimulants, avoid opioids, and have recently experienced a nonfatal fentanyl overdose. We identified multiple mechanisms of overdose from unintentional fentanyl use among our participants. These findings contrast with the idea that overdose from unintentional fentanyl use is a result only of fentanyl contamination of stimulants at the point of manufacture or distribution, which has been a frequently cited mechanism.^16,17,20^ Instead, overdose from unintentional fentanyl use may more often result from the similar appearances of the substances, changes in the drug supply leading to the ubiquity of fentanyl, the frequent social contact between people who primarily use stimulants and those who use opioids, and structural barriers to safety.

Accounts of how these events occur and how people attempt to prevent their occurrence are consistent with the literature on drug, set, and setting influencing overdose risk.^35^ Many of the overdoses involved social mixing of people who use stimulants and avoid fentanyl with people who regularly use fentanyl.

As described by the intersectional risk environment framework, these individual, interpersonal, and situational aspects of drug use behaviors contributing to overdose are influenced by the structural context, including as housing policy, drug policy, and poverty.^32^ Consistent with prior work tracing how oppressed groups including people who use drugs internalize blame and responsibility for structurally created risks, participants often blamed themselves for unintentional fentanyl use.^34,36–38^ We summarize our findings on how pathways to unintentional fentanyl use are constituted at the intersection of individual, interpersonal, and structural factors (Figure).

### 4.1 The Risk Environment and Unintentional Fentanyl Use

It is well documented how socioeconomic status, structural racism, gender power dynamics, stigmatization of people who use drugs, and other forms of marginalization intersect to increase drug-related harms.^39–43^ The intersectional risk environment framework describes how drug use risks are shaped by the drug use environment on the micro (e.g., interpersonal dynamics, drug use setting) and macro (e.g., drug policy, systemic racism) levels.^31,32^ This framework can be applied to unintentional fentanyl use to interrogate how policies and structures influence overdose risk. Among our participants, the ubiquity of fentanyl, financial precarity, housing cost and restrictive policies in city-funded housing, gendered power dynamics, and the lack of access to safer places to use drugs intersected to increase vulnerability to unintentional fentanyl use.

Drug policies at the national and local levels criminalizing drugs and drug use shape the risk of overdose from unintentional fentanyl use, as they do for opioid overdose risk more broadly.^44^ We found that the ubiquity of fentanyl in a changing drug supply has made it harder for people who use stimulants to protect themselves from opioids, and the introduction of fentanyl to the drug supply has created a new risk that did not exist before for people who use stimulants. Prior to fentanyl, black tar heroin was the predominant opioid sold in San Francisco and other West Coast drug markets, and was easily visually distinguishable from stimulants.^45^ Drug prohibition laws focused on targeting heroin suppliers created the market for a more easily synthesized opioid like fentanyl,^46^ and disruptions in the drug market from law enforcement have been shown to increase vulnerability to overdose.^47^

Limited safe spaces for people who use drugs to gather and engage in support services or social activities could reduce the isolation that contributes to overdose risk identified by our participants. Political and legislative barriers exist to implementing supervised consumption sites for overdose prevention, with the only government-sanctioned site in San Francisco closing in 2022.^48^ In the absence of sanctioned safer use environments, some of our participants filled that need by providing a place for people to use drugs in their living space, and others were left using drugs on the street, increasing their vulnerability to unintentional fentanyl use. Creative solutions considering the physical and social environment in overdose prevention are required to address unintentional fentanyl use. To effectively serve people who use stimulants, any such solutions need to be acceptable and accessible to people who smoke in addition to those who inject drugs.^49,50^

Housing and housing policies also played a significant role in shaping the risk of unintentional fentanyl use among our participants, which is consistent with prior literature linking homelessness and unstable housing to increased risk of overdose and other drug-related harms.^51–53^ The SRO environment itself can lead to people using alone and increase overdose risk.^54,55^ Our participants described the restrictive policies within SROs and shelters, including those that surveil participants and prohibit guests, with some naming these policies as the reason why they reduced their own precautions to protect themselves from fentanyl. Housing initiatives that restrict residents’ visitor rights may undermine overdose prevention strategies that rely on bystander response. One promising strategy to counteract high rates of overdose within SROs is a tenant-led response model, in which tenants are trained to be overdose “specialists” who distribute naloxone, perform safety checks on other tenants, and respond to overdoses in the building. The use of wall-mounted “Brave Buttons” that alert overdose specialists to a potential overdose have also shown promise in mitigating risk of overdose death in SROs.^56^ This tenant-led approach has been shown to strengthen mutual-aid practices among SRO tenants, promote autonomy, and enable private but safer drug use,^57^ which might extend to better responses to unintentional fentanyl use overdoses in housing.

In our study, gendered power differentials shaped the risk environment for unintentional fentanyl use among participants identifying as women when they relied on men to procure and prepare their drugs. In the process, they relied on others to differentiate between stimulants and fentanyl when preparing their drugs. This aligns with literature that has shown that gendered violence and power differentials among people who inject drugs are known to increase risk of drug-related harms for women and gender diverse people who rely on others for drug injection.^58–62^ And while these findings from our study were from cisgender women in heterosexual relationships, this gendered violence applies to people of all gender identities.

Finally, systemic racism, particularly for Black Americans, increases drug-related risk for minoritized populations and intersects with the other forms of structural inequity we found influence the risk of unintentional fentanyl use.^63–65^ Across the US, Black Americans are less likely to have access to harm reduction services and addiction care^66–68^ and as community harm reduction services often serve as front-line prevention and education in shifting risk environments, Black Americans may therefore be less prepared to respond to new risks from changes in drug supply, such as unintentional fentanyl use. Our prior research demonstrated that the racial disparities Black San Francisco residents experience in overdose mortality are also seen in unintentional fentanyl use overdose.^13^ While unintentional fentanyl use is unlikely to be the primary contributor of disparity in overdose mortality for Black people, it may be one pathway through which this disparity manifests or is amplified. In the present study we highlight how the risk of unintentional fentanyl use is shaped by other structural inequities including income inequality and housing policy, the intersection of which with structural racism is well documented.^63–65^ Given the overrepresentation of Black Americans in unintentional fentanyl use, cultural adaptation that takes racism into account is needed for individual level interventions to address this phenomenon.^69–71^

### 4.2 Implications for harm reduction practices

Harm reduction supplies, education, and tailored behavioral interventions all have potential to decrease the risk of overdose from unintentional fentanyl use. Given the wide variety of mechanisms leading to unintentional fentanyl use, and no one dominant mechanism, there is no one solution that will prevent unintentional fentanyl use for everyone who uses stimulants.

Distribution of harm reduction supplies including smoking equipment, fentanyl test strips, and naloxone may help prevent overdose from unintentional fentanyl use. In particular, distribution of the glass pipes widely used in San Francisco to smoke stimulants and fentanyl may serve as a strategy to decrease the sharing of equipment, which was identified as a pathway for unintentional fentanyl use. Furthermore, given the overlap in types of glass smoking equipment used for consuming fentanyl and stimulants, the frequent mixing up of pipes might be addressed with pipe markers to allow people to differentiate between pipes that contain a stimulant and pipes that contain fentanyl.

Naloxone distribution is an evidence-based overdose prevention intervention among people who use drugs,^72^ though qualitative research among people who use opioids demonstrate barriers such as fear of legal or social repercussions and uncertainty in when to use naloxone during an overdose.^73,74^ Additional intervention may be needed to promote naloxone uptake among people who use stimulants and not opioids, as they may not perceive a need for naloxone and thus be less likely to carry naloxone and unprepared to respond to an overdose.^75^ Fentanyl test strips are another risk reduction tool that may be helpful for participants who obtain fentanyl while thinking it is a stimulant. While previous qualitative research highlights acceptability of fentanyl test strips among people who use drugs, no trials have demonstrated efficacy in overdose prevention.^76–78^

Limited educational and behavioral interventions exist to prevent opioid overdose among people who use stimulants. Adapting overdose prevention interventions to this population may hold promise for reducing risk. Our participants lacked knowledge on fentanyl and the ways it can appear. Participants used sensory identification of drugs as a main risk reduction method, which is consistent with previous research demonstrating visual drug checking as a primary method used by people who use heroin and people who use both stimulants and opioids.^6,20,79^ However, we were able to uniquely see how this method fails for people who use stimulants and avoid opioids, particularly due to an inability to differentiate between fentanyl and stimulants, and misperceptions of the true appearance of fentanyl.

Education is a potentially effective harm reduction tool^80,81^ that can be used to inform clients of the true difficulty of differentiating between fentanyl and stimulants. Harm reduction practices might be adapted from those deployed by people who use heroin to protect themselves from overdose. For example, “test shots” or “slow shots”^12,82,83^ might be adapted to people who use stimulants to reduce risk of overdose from unintentional fentanyl use. Rigorous evaluation of a combination of possible overdose prevention interventions for this population could generate evidence for public health response. Furthermore, cultural adaptation of prevention interventions for minoritized populations may be an important part of assuring they are responsive to the needs of community disproportionately impacted by unintentional fentanyl use overdose.

### 4.3 Limitations and directions for further research

This paper has several limitations. While qualitative studies are not meant to be generalizable, our study includes a very specific population—i.e., people who use stimulants, and not opioids, in San Francisco, with all but one living in SROs. Furthermore, the study population mostly includes older, cisgender, male-identifying participants and only includes people who speak English. These experiences may differ from those who are young, unhoused, nonbinary, trans, or gender-expansive, or people who speak a language other than English. The drug context of San Francisco may be different from other cities, particularly those on the East Coast; however, ongoing research may shed light on potential mechanisms in other settings and pathways for prevention.^84,85^ Despite these limitations, we were able to uniquely capture events that contributed to overdose from unintentional use by people who survived an overdose. Similar research could more deeply examine how social structures intersect with other forms of marginalization, in particular among people experiencing homelessness, to increase the risk of unintentional fentanyl use.

## 5. Conclusion

There are many causes of unintentional fentanyl use that do not involve contamination of the stimulant supply by fentanyl. Unintentional fentanyl use is often driven by the similar appearance of fentanyl and stimulants, with vulnerability to overdose by unintentional fentanyl use increased by systemic structures like poverty and housing instability. The introduction of fentanyl to the drug supply has created a new overdose risk for people who use stimulants that previously did not exist. Changes to the drug supply arrive in, and can reproduce, a landscape of risk already shaped by historic structural inequities. Our findings highlight how policies and the drug use environment influence the risk of overdose from unintentional fentanyl use and have direct implications for policy change and individual-level interventions to prevent overdose from unintentional fentanyl use.

## Figures and Tables

**Figure 1:**
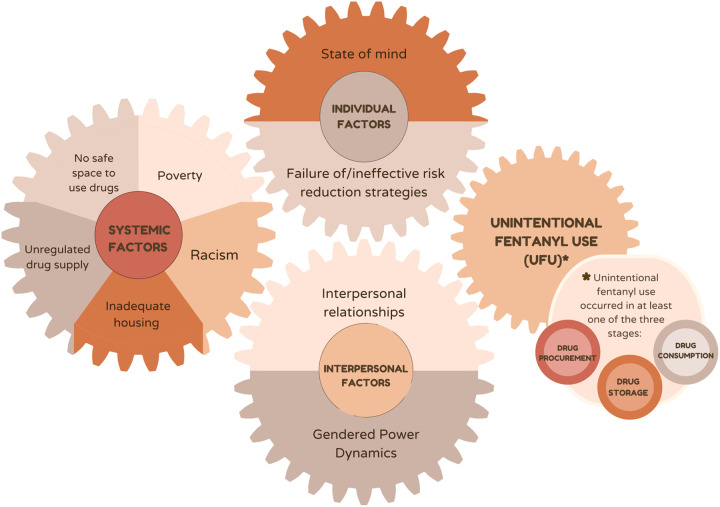
Mechanisms of Unintentional Fentanyl Use

**Table 1:** Characteristics of interview participants who used stimulants and experienced an unintentional fentanyl use overdose (n = 17)

Gender Identity	Count (%)
Male	12 (71%)
Female	5 (29%)
**Age**	
< 30	1 (6%)
30–45	3 (18%)
46–60	8 (47%)
61+	5 (29%)
**Race and Ethnicity** *	
Asian	1 (6%)
Black/African American	9 (52%)
Hispanic/Latinx	7 (41%)
Indigenous/Native American	2 (12%)
White	2 (12%)
**Drug(s) of Choice**	
Only powder cocaine	1 (6%)
Only crack cocaine	3 (18%)
Both powder and crack cocaine	2 (12%)
Only methamphetamine	8 (47%)
Both cocaine and methamphetamine	3 (18%)
**Routes of Administration Used for Stimulants** *	
Injection	6 (35%)
Intranasal	4 (24%)
Smoking	16 (94%)
**Routes of Administration of Drug at Time of Most Recent Overdose**	
Injection	4 (24%)
Intranasal	3 (18%)
Oral	1 (6%)
Smoking	9 (53%)
**Currently Injects Stimulants**	
Yes	6 (35%)
No	11 (65%)
**Lifetime History of Injection Drug Use**	
Yes	9 (53%)
No	8 (47%)
**Housing Status**	
Single-room occupancy (SRO) apartment	16 (94%)
Shelter/unhoused	1 (6%)
**Time From Overdose to Interview**	
Days, median (range)	19 (5–119)

* Categories are not mutually exclusive and can add to greater than 100%

## Data Availability

The qualitative data used for this study are available from the corresponding author with reasonable request and IRB approval.

## Abbreviations

HOPE: Home Outreach Prevention and Engagement
SRO: Single Room Occupancy
